# Modeling directional spatio‐temporal processes in island biogeography

**DOI:** 10.1002/ece3.1632

**Published:** 2015-10-03

**Authors:** José C. Carvalho, Pedro Cardoso, François Rigal, Kostas A. Triantis, Paulo A. V. Borges

**Affiliations:** ^1^ CE3C – Centre for EcologyEvolution and Environmental Changes / Azorean Biodiversity Group and Universidade dos Açores – Departamento de Ciências Agrárias9700‐042 Angra do HeroísmoAçoresPortugal; ^2^ Department of BiologyCBMA – Centre for Molecular and Environmental BiologyUniversity of Minho4710‐087 BragaPortugal; ^3^ Finnish Museum of Natural HistoryUniversity of HelsinkiP.O. Box 17, 00014 HelsinkiFinland; ^4^ Department of Ecology and TaxonomyFaculty of BiologyNational and Kapodistrian UniversityAthens GR‐15784Greece; ^5^ Conservation Biogeography and Macroecology ProgrammeSchool of Geography and the EnvironmentUniversity of Oxford, South Parks RoadOxford OX1 3QYUK

**Keywords:** Community assembly, dispersal limitation, island colonization, network analysis, oceanic islands, spatio‐temporal modeling

## Abstract

A key challenge in island biogeography is to quantity the role of dispersal in shaping biodiversity patterns among the islands of a given archipelago. Here, we propose such a framework. Dispersal within oceanic archipelagos may be conceptualized as a spatio‐temporal process dependent on: (1) the spatial distribution of islands, because the probability of successful dispersal is inversely related to the spatial distance between islands and (2) the chronological sequence of island formation that determines the directional asymmetry of dispersal (hypothesized to be predominantly from older to younger islands). From these premises, directional network models may be constructed, representing putative connections among islands. These models may be translated to eigenfunctions in order to be incorporated into statistical analysis. The framework was tested with 12 datasets from the Hawaii, Azores, and Canaries. The explanatory power of directional network models for explaining species composition patterns, assessed by the Jaccard dissimilarity index, was compared with simpler time‐isolation models. The amount of variation explained by the network models ranged from 5.5% (for Coleoptera in Hawaii) to 60.2% (for Pteridophytes in Canary Islands). In relation to the four studied taxa, the variation explained by network models was higher for Pteridophytes in the three archipelagos. By the contrary, small fractions of explained variation were observed for Coleoptera (5.5%) and Araneae (8.6%) in Hawaii. Time‐isolation models were, in general, not statistical significant and explained less variation than the equivalent directional network models for all the datasets. Directional network models provide a way for evaluating the spatio‐temporal signature of species dispersal. The method allows building scenarios against which hypotheses about dispersal within archipelagos may be tested. The new framework may help to uncover the pathways via which species have colonized the islands of a given archipelago and to understand the origins of insular biodiversity.

## Introduction

On oceanic islands, dispersal and successful establishment are the critical starting processes in the generation of endemic biodiversity, without which diversification within these islands could not take place (e.g., MacArthur and Wilson 1967; Whittaker and Fernández‐Palacios 2007; Whittaker et al. 2008). Although apparently stochastic dispersal patterns have been observed (Wagner and Funk 1995), particularly for species with high dispersal abilities colonizing remote insular systems (Holland and Hadfield 2004), dispersal tends to be a spatially structured process resulting from the interaction between the relative spatial location of islands and the degree of vagility of the taxa considered (Williamson 1981; Paulay 1994; Algar et al. 2013; Carvalho and Cardoso 2014).

Intra‐archipelagic dispersal on volcanic archipelagos depends also on the chronological sequence of the formation of islands, often giving rise to a progression rule pattern, that is, the sequence of colonization of a lineage tends to follow the sequential emergence of islands within an oceanic archipelago and thus corresponds to their respective geological ages (Funk and Wagner 1995; Knox 1999; Whittaker et al. 2008; Eckstut et al. 2011). Thus, intra‐archipelagic dispersal usually is an asymmetric directional process, occurring from older to younger islands. Although back‐colonizations may also occur, they are usually less frequent (e.g., Funk and Wagner 1995; Garb and Gillespie 2009). The progression rule pattern has been observed and established for many taxa in different archipelagos (e.g., Whittaker and Fernández‐Palacios 2007; Cowie and Holland 2008; Gillespie et al. 2008; Parent et al. 2008). Additionally, as predicted by the general dynamic model of oceanic island biogeography (Whittaker et al. 2008), the progression rule should be a common/dominant phylogeographical pattern within any oceanic archipelago in which there is a pronounced age sequence (Whittaker et al. 2008, 2010).

Dispersal within mainly oceanic archipelagos may therefore be conceptualized as a spatio‐temporal process. In order to understand the role of dispersal on the diversity patterns in oceanic volcanic archipelagos, it is necessary to develop a framework that accounts explicitly for two major components: (1) the spatial distribution of islands, because the probability of successful dispersal is negatively related to the spatial distance between islands (Paulay 1994) and (2) the chronological sequence of the formation of islands that determines the directional asymmetry of dispersal (hypothesized to be predominantly from older to younger islands). Although, asymmetric dispersal can be originated by broad‐scale factors, such as prevailing winds, sea currents, and migratory routeways (see Gillespie et al. 2012 and references therein), in this paper, focus on directionality caused by the chronological sequence of island emergence.

Asymmetric Eigenvector Maps (AEM, Blanchet et al. 2008, 2011) is a spatial statistical method to explicitly model the influence of asymmetric directional spatial processes, such as a river network or sea currents, on species distributions or other response variables of interest. The AEM method has also been applied to model time series because the processes associated with time are directional (Legendre and Gauthier 2014). The AEM framework is based on the construction of a directional connectivity matrix denoting the spatial or temporal relationships among sites. This matrix may be weighted according to a predefined function representative of the intensity of the connections (e.g., ease of dispersal). From this matrix, a set of eigenfunctions may be extracted for use as explanatory variables in regression analysis or canonical ordination (Legendre and Legendre 2012). Here, we extend this methodology by integrating the spatial location of oceanic islands and the chronological sequence of their formation in the analysis, thus accounting for the asymmetry of dispersal within archipelagos.

We tested the performance of the framework by modeling the variation in community composition of different biotas accounting for several different taxonomic groups, ferns (Pteridophytes), seed plants (Spermatophytes), spiders (Araneae), and beetles (Coleoptera), among the islands of three oceanic volcanic archipelagos, the Hawaii, Azores, and Canaries. To our knowledge this is the first attempt to integrate eigenfunction analysis explicitly in the context of island biogeography. Our aim is to determine the importance of the directional asymmetry of dispersal in explaining the variation in species composition of insular native biota.

## Materials and Methods

### Building directional network models

**Figure 1:**
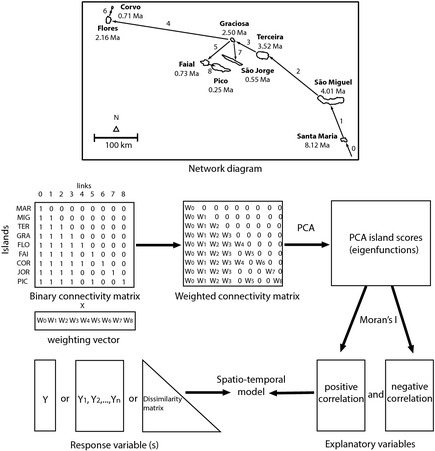
Diagram showing the construction of eigenfunctions representative of the directional spatio‐temporal relationships between islands, illustrated by the Azores. The arrows and numbers represent the links connecting islands according to their chronological sequence of formation and distance among them, using a first nearest neighbor network algorithm. The eigenfunctions correspond to the site scores of a principal components analysis (PCA) carried out on a weighted connectivity matrix (see text for details). These eigenfunctions may be used as independent variables in common statistical analysis to model a response variable, multiple response variables or dissimilarity matrices (compositional or phylogenetic data).

Here, we show how to extend the AEM framework developed by Blanchet et al. (2008) to model directional processes in the context of island biogeography. The neighborhood relationships among the islands of a given archipelago may be represented by a binary connectivity matrix (**C**), where rows are assigned to islands and columns are assigned to the hypothesized connections (links) between islands. This matrix indicates which islands are connected, directly or indirectly, by a given link, by an entry of 1, and those that do not, by 0. The crucial step is to build an algorithm that accounts for the spatial location and the chronological sequence of formation of islands to define the links between them. Here, we define, for the first time, such an algorithm. The algorithm is based on the premise that the connections between islands should be directional, from the older to the younger islands, representing the most probable direction of species movement. The algorithm starts by linking the oldest island (first island to emerge) of a given archipelago to an external source pool (link 0). This link represents the initiation of the colonization process. The second island to emerge is linked to the oldest one (link 1). The third island in the chronological sequence of origin is linked to the nearest of the previous two older islands (link 2). The algorithm proceeds by linking the subsequent islands in the chronological sequence of formation to the nearest older island of the archipelago. The algorithm stops when the youngest island (last island to emerge) is linked to the nearest older island of the archipelago (link *n*‐1, being *n* the number of islands). This simple algorithm produces a first nearest neighbor network linking all the islands of a given archipelago (see Fig. 1 for a graphic display of the neighbor network for the Azores archipelago and the analytical procedures). The first neighbor network represents the well‐known progression rule pattern, characteristic of some archipelagos (Funk and Wagner 1995; Gillespie et al. 2008).

The connectivity matrix can be extended to higher levels of neighborhood to account for the movement of species passing over one or more islands in colonizing the newest island. For example, a younger island may be linked to the two closest older islands. This is the 2nd nearest neighbor network. If we proceed with this rationale, we will end with the most complex model: A younger island may be linked to all the older islands. Therefore, we may have *n*‐1 binary **C** matrices, representing a hierarchy of neighbor networks (1st, 2nd, 3rd … *n*‐1th neighbor network).

Each **C** matrix can be used directly to extract eigenfunctions or may be weighted by a given function, reflecting the ease of dispersal between islands. In this context, Dray et al. (2006) proposed two weighting functions: f1 = 1/*d*_*ij*_^α^ and f2 = 1− (*d*_*ij*_/max(*d*_*ij*_))^α^, where *d*_*ij*_ is the geographic distance between islands *i* and *j* and α is a positive real number. The function f1 is a concave‐up function whereas the function f2 is linear when α = 1 and concave‐down when α > 1. An alternative function to weight the links between islands is:

$$f3=t_{i}^{\beta }/d_{ij}^{\alpha }$$

where *t*_*i*_ is the age of the younger island and *d*_*ij*_ is the geographic distance between the island *i* and its nearest older neighbor *j*. α and β are positive real numbers. Note that the age of the younger island represents the duration of the connection between two islands, as the dispersal of species from an older to a younger island is possible only after the younger one emerges.

Finally, the **C** matrix should be multiplied by the weighting vector in order to obtain a final weighted connectivity matrix (**C**_w_) depicting the spatio‐temporal relationships among the islands of a given archipelago.

The proposed methodology offers a versatile representation of the hypothesized spatio‐temporal relationships among the islands of a given archipelago. Previous knowledge of the archipelago' ontogeny can be incorporated into the network models. For example, models may account for the existence of seamounts that were islands within the time frame of the archipelago in question, by defining links associated with their geographic location. Moreover, ancient connections between islands that formed a single landmass during periods of lowered sea‐level can be accounted for by giving stronger weights to those links. Furthermore, weights may be also calculated using distances among islands based on lowered sea levels.

### Transforming directional network models into eigenfunctions

The next step is to transform the **C**_w_ matrix (or **C** if no weights were applied) into eigenfunctions for use as explanatory variables in regression or canonical analysis. The simplest way to build eigenfunctions is to carry out a principal components analysis (PCA) of the **C** or **C**_w_ matrices. The PCA island scores correspond to the eigenfunctions (AEM eigenfunctions in the terminology of Blanchet et al. 2008). The eigenfunctions may be plotted in geographic maps to aid in their interpretation. Alternatively, one could also plot the fitted values of statistical models built with the eigenfunctions as predictors in geographic maps. Note that other equivalent approaches to producing eigenfunctions are also possible (see Blanchet et al. 2008; Legendre and Legendre 2012).

Usually, *n*‐1 eigenfunctions are produced (where *n* is the number of islands). These eigenfunctions are orthogonal to each other and can be used as independent explanatory variables. Another characteristic of the eigenfunctions is that they model positive and negative autocorrelation. To separate the eigenfunctions into two sets of positive and negative autocorrelation, the Moran's *I* coefficient may be calculated. If the observed value is higher than the expected *I*‐value under the null hypothesis of an absence of autocorrelation, the eigenfunction is deemed to represent positive autocorrelation; otherwise, it represents negative autocorrelation (Gittleman and Kot 1990). In general, one is more interested in positive autocorrelation originated by contagion processes due to species dispersal. Therefore, the positively correlated eigenfunctions can be used as explanatory variables in statistical models against a response variable (e.g., multiple regression), a sites × species table (e.g., redundancy analysis), or a dissimilarity matrix (e.g., distance‐based redundancy analysis).

### Selecting the “best” network model

Given a hierarchy of network models of increasing neighborhood, the next question is which one best represents the process being studied (e.g., colonization). In the absence of a clear a priori hypothesis, one may consider the full range of network models and select the one that explains more variation of the biological phenomenon of interest, or the most parsimonious model based on variable selection procedures (see Legendre and Legendre 2012; for a discussion on several approaches) and information theoretic procedures (Burnham and Anderson 1998). However, one should be aware that higher order networks had little additional information than simpler ones. This happens because as the neighborhood order of networks increases, the number of new added links diminishes. Therefore, in practice one may consider only the first few neighbor networks in the hierarchy.

We envisage that the best strategy to explore network models is to define a priori the degree of neighborhood to consider as determined by the particular hypothesis being tested. For example, one may wish to test the effects of a directional stepping‐stone colonization model, which is represented by the first nearest neighbor network.

Another issue to consider is the weighting scheme for the links between islands. In this context, the weights may be dependent on the complexity of the geological formation and geographic structure of the archipelagos. Some archipelagos originate from stationary thermal plumes beneath tectonic plates forming volcanic islands as the plates drift (“hotspot hypothesis”; Wilson 1963). This mechanism usually gives rise to a linear chain of islands oriented in the direction of the plate movement following an age progression (e.g., Hawaii, Australs, and Marquesas). Other archipelagos are associated with the boundaries of tectonic plates, such as those forming near mid‐oceanic ridges (e.g., Azores, Iceland), or along a subduction zone (e.g., Solomon, Tongan Islands). Such archipelagos usually do not show a clear linear relationship between their spatial distribution and their ages of emergence. Thus, for hotspot archipelagos, we hypothesize that unweighted connectivity networks could be expected to perform better, while for other types of archipelagos, such as the Azores, more complex weighted networks may be required to model island biodiversity patterns. In this case, several weighting schemes could be tested in order to find the proper weights for the links between the islands.

### Case studies

**Figure 2:**
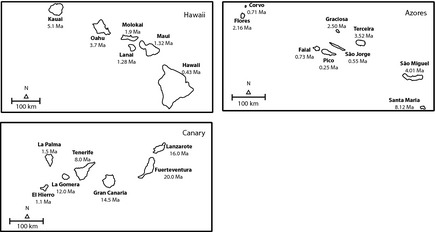
Maps of the selected archipelagos with geological ages indicated (see Appendix S1 for references).

A crucial challenge in insular biogeography is to understand the processes responsible for the assembly of communities on islands. We hypothesize that directional effects have an important role in shaping community composition variation among islands. To test this hypothesis, we applied the directional network modeling approach to 12 datasets consisting of exhaustive checklists of the distribution of ferns (Pteridophytes), seed plants (Spermatophytes), spiders (Araneae), and beetles (Insecta, Coleoptera) across the Hawaii, Azores, and Canary Islands archipelagos (Fig. 2). Introduced species, subspecies, and varieties were excluded from the analysis. The principal data sources and a summary of biological and geographical data are provided on Appendix S1 in Supporting Information.

### Statistical analysis

The variation in species composition between islands was quantified by the Jaccard pairwise dissimilarity index (Jaccard 1912). The resulting dissimilarity matrices (β‐matrices) were used as the responses to build statistical models, for each taxon in each archipelago, by means of distance‐based redundancy analysis (dbRDA – Legendre and Anderson 1999).

#### Directional network models

To model spatio‐temporal variation, we considered the simplest first‐order network model for each archipelago, corresponding to a hypothesized predominant directional stepping‐stone mode of dispersal. The functions f1, f2, and f3, with α = 2 and β = 1, were considered to weight the links. We also considered networks without weights (link present = 1; link absent = 0). In order to select the “best” weighting scheme for each archipelago, for the four taxa in conjunction, we performed a congruence among distance matrices (CADM) analysis (Legendre and Lapointe 2004; Campbell et al. 2011). First, each **C**_w_ matrix (or **C** when no weights were applied) was transformed into a Euclidean pairwise distance matrix (**C**_E_). Hence, we obtained four candidate distance matrices, for each archipelago, derived from: (1) unweighted networks; (2) weighted networks with the function f1; (3) weighted networks with the function f2; and (4) weighted networks with the function f3. Second, the Kendall's *W* concordance statistic was calculated between each candidate distance matrix and the set of four β‐matrices in each archipelago. The *W* statistic provides an estimate of the degree of congruence on a scale between 0 (no congruence) and 1 (complete congruence) among the matrices. Values were tested for significance by permutation (999 permutations), although this is not strictly necessary as we were only interested in selecting the best weighting scheme and not in testing a particular hypothesis. For each archipelago, we selected the weighting scheme that provided the highest congruence (highest *W*) among the **C**_E_ matrix and the set of four β‐matrices.

#### Simulation study

We further test the validity of the first nearest neighbor network models by comparing the observed *W* coefficient with those obtained with simulations of random networks, taken the four taxa in conjunction. We generated 999 connected directional networks with the Erdős–Rényi random model (Erdős and Rényi 1959) for each archipelago and calculated the Kendall's *W* coefficient among the **C**_E_ matrices of these networks and the set of four β‐matrices, as mentioned above. This allowed obtaining a null distribution of *W* values. The simulations were based on directional random networks that preserve the number of islands and the number of links used in the first nearest neighbor network models, but not the chronological sequence of island formation, in order to exclude the mechanism being tested.

#### Modeling the spatio‐temporal variation in species composition

For each first nearest neighbor network model, we therefore performed the PCA and we run Moran's I to retain the eigenfunctions that represented only positive autocorrelation to be used as explanatory variables in a dbRDA with each β‐matrix as the response. In order to select the most parsimonious subset of eigenfunctions that best explain the variation of each β‐matrix, we used the adjusted coefficient of determination ($R_{a}^{2}$) as a measure of explained variation (Peres‐Neto et al. 2006). We did not use AICc as a model selection criterion because the response in the models is a dissimilarity matrix. However, it is worth noting that the $R_{a}^{2}$ adjusts for the number of variables and observations in the models. We tested all possible combinations of eigenfunctions and we selected the subset, with all of their terms significant, that explain together the largest proportion of variation of the β‐matrix (larger $R_{a}^{2}$). Tests of significance were carried out by permutation. A statistical significance of 0.1 was set for these analyses, as significance levels that were more restrictive could hide important but less strong relationships due to the small number of islands considered for each archipelago.

#### Comparing the performance of network models with simpler time‐isolation models

For each archipelago and taxa, the performance of directional network models was compared with a simpler time‐isolation model including the raw variables that were used to build the networks as explanatory variables instead of the eigenfunctions: the age of islands (Time) and the distance from a younger island to its nearest older neighbor (Dnold). Note that these time‐isolation models are equivalent to the network models, without considering explicitly the putative directional links among the islands. Therefore, the comparison between both approaches is valid.

Analyses were performed in the R environment (R Core Team, 2014) using the packages: ape (Paradis et al. 2004) for CADM analysis, igraph (Csardi and Nepusz 2006) to create random networks, AEM (Blanchet and Legendre 2012) to build eigenfunctions and test their autocorrelation, and vegan (Oksanen et al. 2013) to carry out dbRDA.

## Results

### Directional network models

#### Selecting the best weighting scheme for the networks

For each archipelago, we identified the weighting scheme for the first nearest neighbor network model by CADM analysis, taken the four taxa in conjunction. For the Hawaii and Canary Islands, binary connectivity networks and the set of four β‐matrices showed the highest congruence (*W* = 0.660, *P* = 0.001 and *W* = 0.840, *P* = 0.001, respectively). For the Azores, the network weighted by the function f3 in conjunction with the β‐matrices had the highest congruence (*W* = 0.551, *P* = 0.001). Therefore, these networks were retained for further analyses.

#### Simulation study

**Figure 3:**
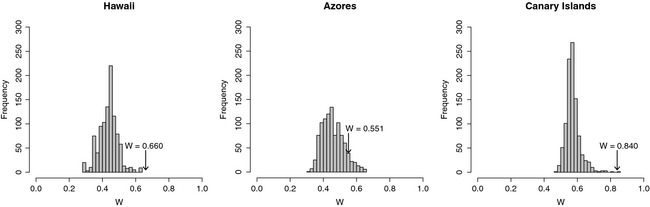
Frequency distribution of Kendall's *W* coefficient of concordance calculated for 999 generated Erdős–Rényi random networks and the set of four β‐matrices (Pteridophytes, Spermatophytes, Araneae, and Coleoptera) across Hawaii, Azores, and Canary Islands (see text for details). The *W* values calculated for directional network models are shown by an arrow.

The *W* values calculated for the first nearest neighbor network models were compared with those obtained from 999 simulations of directional random networks built for each archipelago (Fig. 3). In the cases of Hawaii and Canaries, no weights were applied to the links, while for the Azores the random networks were weighted by the function f3, as this was the best weighting scheme for the first nearest neighbor network model. The average *W* coefficient obtained with simulations was 0.436 (SD = 0.056) for Hawaii, 0.457 (SD = 0.065) for Azores, and 0.573 (SD = 0.066) for Canary Islands. The *W* coefficient of the directional networks was greater than all of those of the random networks for Hawaii, 913 of the random networks for Azores and 997 of the random networks for Canary Islands. These results provide substantial support in favor of directional network models.

#### Modeling the spatio‐temporal variation in species composition

From the first nearest neighbor networks, two eigenfunctions were retained for Hawaii, while four eigenfunctions were selected for Canaries and Azores. For each dataset, we tested different combinations of eigenfunctions and selected the model, with partial significant terms (α = 0.1), that explained the largest amount of variation in species composition in terms of $R_{a}^{2}$ values, by dbRDA (Table 1). The amount of variation explained by the network models ranged from 5.5% (for Coleoptera in Hawaii) to 60.2% (for Pteridophytes in Canary Islands). In relation to the four studied taxa, the variation explained by network models was higher for Pteridophytes for the three archipelagos. In contrast, small fractions of explained variation were observed for Coleoptera and Araneae for Hawaii.

**Table 1:** Directional network models explaining community composition variation (measured as the Jaccard dissimilarity index) of Pteridophytes, Spermatophytes, Araneae, and Coleoptera from Hawaii, Azores, and Canaries. The eigenfunctions (*X*1, *X*2,…, *Xn*) were obtained from first nearest neighbor networks (see Fig. 3 for a geographical representation of the selected eigenfunctions). The variation explained by each model is expressed in terms of $R_{a}^{2}$ (%). *P*‐values refer to the significance of the global model, and each variable was significant at the α = 0.1

Archipelago	Taxon	Model	$R_{a}^{2}$	F	*P*
Hawaii	Pteridophytes	*X*1 + *X*2	41.0	2.737	0.007
	Spermatophytes	*X*1 + *X*2	23.4	1.764	0.005
	Araneae	*X*1	8.6	1.470	0.032
	Coleoptera	*X*1 + *X*2	5.5	1.146	0.031
Azores	Pteridophytes	*X*4	27.0	3.954	0.022
	Spermatophytes	*X*4	14.5	2.353	0.046
	Araneae	*X*1	18.6	2.825	0.061
	Coleoptera	*X*1	16.3	2.560	0.003
Canary Islands	Pteridophytes	*X*1	60.2	10.057	0.025
	Spermatophytes	*X*1	31.8	3.797	0.009
	Araneae	*X*1	18.5	2.365	0.005
	Coleoptera	*X*1	24.4	2.938	0.009

**Figure 4:**
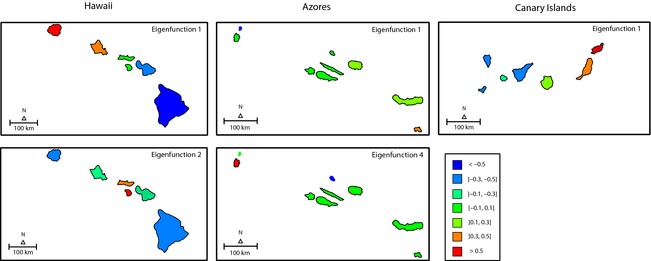
Geographical representation of the selected eigenfunctions used to build directional network models for Pteridophytes, Spermatophytes, Araneae, and Coleoptera across Hawaii, Azores, and Canary Islands. The eigenfunctions (*X*1, *X*2,…, *Xn*) were obtained from first nearest neighbor networks. It should be noted that eigenfunctions can be interpreted as the island scores extracted from a PCA carried out on the connectivity matrix. The Azores islands were enlarged in relation to the map scale to facilitate visualization.

The interpretation of eigenfunctions is straightforward, as they actually are the island scores of a PCA analysis carried on the **C** or **C**_w_ matrices. Therefore, islands with different signs (positive vs. negative scores) represent patterns of differentiation, while islands with similar scores represent patterns of similarity (Fig. 4). In Hawaii, for Spermatophytes, Pteridophytes, and Coleoptera, the eigenfunction 1 displays a gradient showing the directional process corresponding to the chronological sequence of formation of islands. The eigenfunction 2, selected for the four taxa, corresponds to the connection between Molokai and Lanai, which is the only link that does not follow the age progression and linear geographic distribution characteristic of the Hawaii archipelago.

In the Azores, the eigenfunction 1, selected for Coleoptera and Araneae, represents a progression from Santa Maria to Terceira (positive scores) and the isolation of Corvo (negative score). The central group of islands and Flores were grouped together (scores near zero). The eigenfunction 4, selected for Spermatophytes and Pteridophytes, mainly differentiates Graciosa from Flores, two consecutive islands in the chronological sequence, but which are distant from one another. The patterns for the Azores illustrate the likely influence of geographic distance in promoting the differentiation of communities between islands that tended to follow an age progression but that are quite distant from each other.

In the Canary Islands, the eigenfunction 1 was selected for the four taxa. This eigenfunction represents a pattern of differentiation into two opposite directions, from Fuerteventura to Lanzarote and from Fuerteventura to Gran Canaria, La Gomera and the remaining islands. Tenerife, La Palma and El Hierro were grouped together (had equal scores), indicating a low differentiation among them.

### Comparing the performance of network models with simpler time‐isolation models

The construction of directional network models was based on two basic variables: the age of islands and the distance of a given island to its nearest older neighbor. Models including these raw variables were, in general, not statistical significant and explained less variation than the equivalent directional network models for all the datasets (Table 2). Because both models used the same variables, these results testify the importance of considering directionality in model building for oceanic archipelagos.

**Table 2:** Time‐isolation models explaining community composition variation (measured as the Jaccard dissimilarity index) of Pteridophytes, Spermatophytes, Araneae, and Coleoptera from Hawaii, Azores, and Canary Islands. The variation explained by each model is expressed in terms of $R_{a}^{2}$ (%). Abbreviations refer to the maximum geological age (Time) and distance to the nearest older neighbor (Dnold). *P*‐values refer to the significance of the global model. Symbols represent the individual significance of each term in the model (ns – not significant; ^(·)^ – 0.1; * – 0.05; ** – 0.01; *** – 0.001)

Archipelago	Taxon	Model	$R_{a}^{2}$	F	*P*
Hawaii	Pteridophytes	Time*+Dnold^ns^	16.9	1.509	0.109
	Spermatophytes	Time**+Dnold^ns^	17.0	1.514	0.028
	Araneae	Time^ns^+Dnold^ns^	0.6	1.015	0.459
	Coleoptera	Time^ns^+Dnold^ns^	1.3	1.032	0.339
Azores	Pteridophytes	Time^ns^+Dnold^ns^	16.4	1.783	0.152
	Spermatophytes	Time^ns^+Dnold^ns^	1.7	1.068	0.415
	Araneae	Time^ns^+Dnold^ns^	<0	0.902	0.507
	Coleoptera	Time^ns^+Dnold^ns^	<0	0.789	0.769
Canary Islands	Pteridophytes	Time^ns^+Dnold^ns^	39.2	2.934	0.145
	Spermatophytes	Time^(·)^ +Dnold^ns^	23.7	1.931	0.085
	Araneae	Time^(·)^+Dnold^ns^	16.5	1.591	0.073
	Coleoptera	Time^(·)^+Dnold^ns^	17.7	1.646	0.118

## Discussion

### Directional network models in island biogeography

The dynamics of colonization of islands is the critical initiation step that ultimately determines the biodiversity patterns within an archipelago. The evidence suggests that directional colonization events, within oceanic archipelagos, are a general trend in island biogeography (Cowie and Holland 2006; Whittaker et al. 2008). As advocated in this paper, directional dispersal may be determined by a spatio‐temporal interaction, as the geological age of islands is one of the critical factors that determines the direction of intra‐archipelagic colonization (see also Borges and Brown 1999; Bonacum et al. 2005; Sequeira et al. 2008) and geographic distance determines the ease of dispersion (Paulay 1994; Carvalho and Cardoso 2014). The framework proposed here allows us to test hypotheses concerning the role of directional dispersal in the establishment of biodiversity patterns on oceanic archipelagos, thus providing a more complete understanding of biogeographic processes.

Some hotspot volcanic archipelagos, such as Hawaii, Australs, and Marquesas, are arranged linearly by the age of emergence of islands. In such archipelagos, colonization patterns may follow a progression rule, reflecting the successive colonization of islands in the order of their formation (Funk and Wagner 1995; Gillespie et al. 2008). The colonization pattern can be less evident in archipelagos with a more complex geological history, such as the Azores (Amorim et al. 2012), Canaries (Sanmartín et al. 2008), Cape Verde (Carranza et al. 2001), or Galapagos (Sequeira et al. 2008). However, as we show here, the careful selection of weights for the links in the networks can reveal more complex patterns than the classic hotspot scenario, for example, for the Azores.

Network models have the potential to be applied in many oceanic island archipelagos as it requires only two basic types of information: (1) the geographic coordinates of the islands and (2) the age of their geological formation. The framework is particularly suitable for volcanic archipelagos because they have a well‐defined chronological sequence of island formation. However, there are some limitations to its use. First, the overall approach can be characterized as “static” since geographical and geological data in terms of the island's ages exist for current, extant islands, and thus, the dynamic nature of the islands and the archipelagos overall cannot be taken into account (see Whittaker et al. 2008, 2010; Fernández‐Palacios et al. 2011; see also Triantis et al. 2015). Additionally, for many oceanic archipelagos, the age of islands emergence is unknown or subject to intensive debates between volcanologists. This limitation could be minimized if the chronological sequence is known despite the actual geological ages being unknown.

Second, the proposed framework does not account for back colonization processes, from younger islands to older ones. In cases where back colonization events might be important (e.g., Kvist et al. 2005), a nondirectional spatial modeling approach would be more reasonable (see Dray et al. 2006). Another approach could be to construct a directional model based on the algorithm provided in this paper and a nondirectional model and separate their influence on the response variable by variation partitioning (Blanchet et al. 2011). Nevertheless, the colonization from older to younger islands should be the dominant process in the majority of volcanic oceanic archipelagos (e.g., Wagner and Funk 1995; Cowie and Holland 2006; Whittaker et al. 2008). For nonvolcanic archipelagos, it is difficult to hypothesize a direction in intra‐archipelagic dispersal. In this case, network models based on nondirectional methods could be more suitable (see Dray et al. 2006).

In this paper, we focus on directional colonization induced by the hypothesized chronological sequence of island formation. Asymmetric dispersal may also be determined by other processes, such as prevailing winds, ocean currents or mediated by migrating birds (see Gillespie et al. 2012 and references therein). These asymmetric dispersal processes differ from those induced by the sequential formation of islands in that they only include a spatial dimension, while the latter includes both spatial and temporal dimensions. In this context, different network models may be built based on different hypothesized directional processes. Their relative importance in explaining a given response may then be directly compared or disentangled through variation partitioning. For example, spatio‐temporal variation may be assessed using the algorithm provided in this paper and then dissected from the variation explained by a network model representing a given hypothetical physical process (e.g., prevailing winds).

### The cases of Hawaii, Azores, and Canaries archipelagos

The framework here provided allowed to improve our knowledge on how community assembly of native invertebrate flora and fauna was shaped for Hawaiian, Azorean, and Canarian archipelagos. We have shown that directional spatio‐temporal effects can explain a significant proportion of variation of community composition for most of the studied datasets. Comparatively to simpler time‐isolation models, directional networks performed much better. This provides evidence that the chronological sequence of appearance of islands and their spatial location exerted an important role in shaping species distributions.

According to our hypothesis, the patterns exhibited by the corresponding eigenfunctions differed among archipelagos as expected. For Hawaii, the selected eigenfunctions suggest the influence of a linear spatial structure on community composition variation, consistent with a predominant stepping‐stone mode of dispersal from older to younger islands in the chain (Funk and Wagner 1995; Cowie and Holland 2006, 2008). Nevertheless, the variation explained by network models for Araneae and Coleoptera was very low. These results may be due to confounding effects caused by species bypassing the colonization sequence or as a result of back colonization events, that is, species colonizing older islands from younger ones (Garb and Gillespie 2009).

For the Canary Islands, although the patterns exhibited by the corresponding eigenfunctions were consistent with a directional mode of dispersal from older to younger islands, they reveal a more complex structure in colonization processes than the stepping‐stone model characteristic of the Hawaiian archipelago. This can be assigned to the longer history, the more complex volcanic activity, the nonlinear geographic distribution of islands and the closer proximity to the mainland of the Canary Islands (Juan et al. 2000; Sanmartín et al. 2008).

For the Azores, although the results were consistent with a directional mode of dispersal from older to younger islands, the simple progression rule pattern seems to be less clear. This may be due to two reasons. First, contrary to the other archipelagos, the formation of Azorean Islands was not linear in space. In fact the two Western Islands are in another tectonic plate from the remaining seven, and the central islands have not formed in strict geographical sequence and are located over a complex micro‐plate. Additionally, the oldest island of the archipelago, that is Santa Maria, was isolated for at least 4 Ma after its formation, before any other of the Azorean islands emerged and in total, 62% of the current Azorean land is younger than 1 Ma old (see related discussion in Triantis et al. 2012). These may have caused more complex colonization patterns, such as extensive bypassing of intermediate islands in the chronological sequence of many clades. Second, the Azores have suffered extensive native forest destruction in the last six centuries, causing many unrecorded extinctions (Cardoso et al. 2010; Triantis et al. 2010; Terzopoulou et al. 2015), which may confound biogeographical analysis for many taxa. Changes in wind circulation may also account for some patterns in current species compositions in the islands.

For Hawaii and Canary Islands, the “best” networks were based on unweighted connectivity matrices, suggesting that the chronological sequence was important for their colonization but the distance between successive islands played a minor role. By the contrary, for Azores weighted networks performed better, which reflects the more clustered spatial structure of Azores islands and the isolation of the western group (Flores and Corvo). Furthermore, the differences of results between archipelagos are evidence for the usefulness of testing different weighting schemes when building network models for archipelagos with a more complex geological history and a clustered geographical structure.

### Future perspectives

Oceanic archipelagos being distinct at spatial and evolutionary scales present opportunities for holistic analyses in biogeography and ecology (Triantis et al. 2015). The use of directional network models provides a hypothesis‐testing framework to forecast the effects of the chronological sequence of island formation on island biodiversity patterns in its multiple facets. Within this framework, we may test hypotheses concerning the role of asymmetric dispersal on the relative diversity and community assembly in oceanic islands. In this context, we envisage that eigenfunctions resulting from directional network models can also be used as explanatory variables against phylogenetic and functional dissimilarity matrices (e.g., Cardoso et al. 2014).

Phylogeographic analysis often provides evidence for the dispersal from older to younger islands within an archipelago, with speciation occurring in newly colonized islands (see Funk and Wagner 1995; Cowie and Holland 2006, 2008 for several examples). Spatial processes may be better retrieved from phylogenetic data than using species lists alone, as the distances between islands and their age should be reflected in the phylogenies of taxa. The framework here presented may, however, allow us to explicitly model the directional process of colonization and consequent speciation. For example, one may aim to disentangle the spatio‐temporal effects, induced by the sequence of island emergence and their spatial distribution, from other island properties, on the genetic divergence patterns among islands. Thus, the framework here provided can be complementary to phylogeographic analyses.

As for functional data, this framework provides a way to model and test hypotheses concerning directional dispersal within archipelagos for taxa with different life‐history traits. For example, it may allow assessment of the role of dispersal limitation among subsets of organisms with different dispersal abilities on island colonization patterns and community assembly (Carvalho and Cardoso 2014).

In conclusion, the strength of the methodological approach presented in this paper was to provide a way to incorporate spatio‐temporal relationships among islands into common statistical models and quantify their effects on community composition patterns, allowing us to go beyond a simple observation of a directional effect. The framework can be easily adapted also for phylogenetic and functional data. Therefore, we advocate that the incorporation of network models in island biogeography is a definitely useful addition to the biogeographer's toolkit.

## Conflict of Interest

None declared.

## Supporting information


**Appendix S1.** The principal data sources and summary of biological and geographical data for Hawaii, Azores and Canary Islands.
