# Supplementary material for: Modeling directional spatio‐temporal processes in island biogeography
Source: Ecol Evol. 2015 Oct 3;5(20):4671–82. doi: 10.1002/ece3.1632 (PMC4670066; doi:10.1002/ece3.1632)
Supplement: Supplementary file 1 — Appendix S1. The principal data sources and summary of biological and geographical data for Hawaii, Azores and Canary Islands. [file ECE3-5-4671-s001.doc]

**SUPPORTING INFORMATION**

Modelling directional spatio-temporal processes in island biogeography

José C. Carvalho, Pedro Cardoso, François Rigal, Kostas A. Triantis, Paulo A. V. Borges

***Ecology and Evolution***

**Appendix S1** This appendix contains the principal data sources and a summary of biological and geographical data for Hawaii, Azores and Canary Islands (Table 1).

*Hawaii*

The Hawaiian archipelago is formed by eight main islands located in the middle of the Pacific Ocean. For our analyses we used data for only 6 islands (see main text, Fig. 2). Kahoolawe Island was excluded from the analysis because it was long used as a testing ground for military activities, and has also harboured a high population of alien ungulates, resulting in an extremely disturbed and non-representative ecosystem. Niihau is a private island and was excluded due to the lack of available data (as e.g. Whittaker *et al*., 2008). Maui, Molokai, Lanai and Kahoolawe formed a single, large island named Maui Nui that reached its maximum areal extent around 1.2 Ma. During Pleistocene glaciations these islands divided and reunited periodically following sea level changes (Price & Elliott-Fisk, 2004). Data for plants were extracted from Imada (2012). For spiders and Coleoptera, data were compiled from Nishida (2002). Spider data were further updated from numerous sources (e.g., Gillespie, 2002; Hormiga, 2002; Gillespie & Rivera, 2007; Proszynski, 2007). The ages of the islands used were as reported in Cowie & Holland (2008).

*Azores*

The Azores archipelago is formed by nine main islands and lies in the Northern Atlantic Ocean (see main text, Fig. 2). The islands form three groups: the eastern (Santa Maria and São Miguel), central (Faial, Pico, Graciosa, São Jorge and Terceira) and western (Corvo and Flores) groups. The islands of Faial and Pico made a single landmass, called Laurinsula, between 30 and 14 ka, 57 and 43 ka and 72 and 66 ka (França *et al*., 2003; Rijsdijk *et al*., 2014). In general, the Azores is considered as an ecologically homogeneous system with wet and mild climate, harbouring a fair proportion of endemic fauna and flora (Triantis *et al*., 2012). The most recent compilation of Azorean fauna and flora (Borges *et al*., 2010) with some updates performed by one of us (PAVB) was used for the extraction of data on the distribution of species and the geological ages of the islands.

*Canary Islands*

The Canarian archipelago is formed by seven main islands that share biotic affinities predominantly with the Mediterranean region (see main text, Fig. 2). All the islands are volcanically active, except La Gomera. Lanzarote and Fuerteventura formed a single landmass (Mahan) between 77 and 9 ka (Rijsdijk *et al*., 2014). The Canaries have an outstanding diversity of major ecosystem types, which coupled with its long history and isolation from the mainland have enabled the development and persistence of a substantial endemic fauna and flora (Reyes-Betancort *et al*., 2008). Data on the distribution of species were extracted from Arechavaleta *et al*. (2010). The ages of the islands used were as reported in Whittaker & Fernández-Palacios (2007) and Fernández-Palacios *et al*. (2011).

**Table 1** Summary of biological and geographic data for the Hawaii, Azores and Canary Islands archipelagos. Minimum and maximum values are provided for each archipelago with corresponding islands in parenthesis.

|  | Hawaii | Azores | Canary Islands |
| --- | --- | --- | --- |
| Nº islands | 6 | 9 | 7 |
| Area (Km^2^) | 361 (Lanai)  10458 (Hawaii) | 17 (Corvo)  750 (São Miguel) | 278 (Hierro)  2058 (Tenerife) |
| Elevation (m) | 1026 (Lanai)  4205 (Hawaii) | 398 (Graciosa)  2351 (Pico) | 670 (Lanzarote)  3711 (Tenerife) |
| Age (Ma) | 0.43 (Hawaii)  5.10 (Kauai) | 0.25 (Pico)  8.12 (Santa Maria) | 1.1 (Hierro)  20 (Fuerteventura) |
| Pteridophytes | 109 (Lanai)  148 (Maui)  187 (total) | 22 (Graciosa)  46 (Pico)  51 (total) | 15 (Lanzarote)  43 (Tenerife)  49 (total) |
| Spermatophytes | 252 (Lanai)  489 (Kauai)  1027 (total) | 75 (Graciosa)  141 (São Miguel)  171 (total) | 517 (Lanzarote)  929 (Tenerife)  1330 (total) |
| Araneae | 14 (Lanai)  70 (Oahu, Maui)  169 (total) | 13 (Corvo)  31 (Terceira)  43 (total) | 85 (Lanzarote, Hierro)  229 (Tenerife)  426 (total) |
| Coleoptera | 129 (Lanai)  453 (Oahu)  1294 (total) | 19 (Corvo)  131 (São Miguel)  199 (total) | 416 (Lanzarote)  1134 (Tenerife)  1923 (total) |

**References**

Arechavaleta, M., Rodríguez, S., Zurita, N. & García, A. (coord.) (2010) *Lista de especies silvestres de Canarias. Hongos, plantas y animales terrestres. 2009*. Gobierno de Canarias, 579 pp.

Borges, P.A.V., Costa, A., Cunha, R., Gabriel, R., Gonçalves, V., Martins, A.F., Melo, I., Parente, M., Raposeiro, P., Rodrigues, P., Santos, R.S., Silva, L., Vieira, P. & Vieira, V. (Eds.) (2010). *A list of the terrestrial and marine biota from the Azores*. Princípia, Cascais, 432 pp.

França, Z., Cruz, J.V., Nunes, J.C. & Forjaz, V.H. (2003) Geologia dos Açores: uma perspectiva actual. *Açoreana*, **10**, 1–140.

Gillespie, R.G. (2002) Hawaiian spiders of the genus *Tetragnatha*: IV New, small species in the spiny leg clade. *Journal of Arachnology*, **30**, 159-172.

Gillespie, R.G. & Rivera. M.A.J. (2007) Free-living spiders of the genus *Ariames* (Araneae, Theridiidae) in Hawaii. *Journal of Arachnology*, **35**, 11-37.

Hormiga, G. (2002) *Orsonwelles*, a new genus of giant linyphiid spiders (Araneae) from the Hawaiian Islands. *Invertebrate Systematics*, **16**, 369-448.

Imada, C. (2012) Hawaiian native and naturalized vascular plants checklist (December 2012 update). *Bishop Musem Technical Report*, **60**, 1-380.

Nishida, G. (2002) Hawaiian terrestrial arthropod checklist. *Bishop Museum Technical Report*, **22**, 1-313.

Price, J. & Elliott-Fisk, D. (2004) Topographic history of the Maui Nui complex, Hawai'i, and its implications for biogeography. *Pacific Science*, **58**, 27-45.

Proszynski, J. (2007) A survey of *Havaika* (Aranei: Salticidae), an endemic genus from Hawaii, including descriptions of new species. *Arthropoda Selecta*, **16**, 195-213.

Reyes-Betancort, J.A., Santos-Guerra, A., Guma, I.R., Humphries, C.J., Carine, M.A. (2008) Diversity, rarity and the evolution of the Canary Islands endemic flora. *Anales Jardín Botánico de Madrid*, **65**, 25–45.

Rijsdijk, K.F., Hengl, T., Norder, S.J., Otto, R., Emerson, B.C., Ávila, S.P., López, H., van Loon, E., Tjørve, E. & Fernández‐Palacios, J.M. (2014). Quantifying surface‐area changes of volcanic islands driven by Pleistocene sea‐level cycles: biogeographical implications for the Macaronesian archipelagos. *Journal of Biogeography*, **41**, 1242-1254.

Whittaker, R.J., Triantis, K.A. & Ladle, R.J. (2008) A general dynamic theory of oceanic island biogeography. *Journal of Biogeography*, **35**, 977–994.
